# Phenotype and genotype heterogeneity of PLA2G6-associated neurodegeneration in a cohort of pediatric and adult patients

**DOI:** 10.1186/s13023-023-02780-9

**Published:** 2023-07-05

**Authors:** Ali Zare Dehnavi, Maryam Bemanalizadeh, Seyyed Mohammad Kahani, Mahmoud Reza Ashrafi, Mohammad Rohani, Mehran Beiraghi Toosi, Morteza Heidari, Sareh Hosseinpour, Behnam Amini, Shaghayegh Zokaei, Zahra Rezaei, Hajar Aryan, Man Amanat, Hassan Vahidnezhad, Pouria Mohammadi, Masoud Garshasbi, Ali Reza Tavasoli

**Affiliations:** 1 Department of Pediatrics, Division of Pediatric Neurology, Children’s Medical Center, Pediatrics Center of Excellence, Tehran University of Medical Sciences, Tehran, Iran; 2 Child Growth and Development Research Center, Research Institute for Primordial Prevention of Non-Communicable Disease, Isfahan University of Medical Sciences, Isfahan, Iran; 3 Faculty of Medical Sciences, Department of Medical Genetics, Tarbiat Modares University, Tehran, Iran; 4 Skull Base Research Center, The Five Senses Health Institute, Rasoul Akram Hospital, Iran University of Medical Sciences, Tehran, Iran; 5 Department of Neurology, Rasoul Akram Hospital, Iran University of Medical Sciences, Tehran, Iran; 6 Department of Pediatrics, School of Medicine, Mashhad University of Medical Sciences, Mashhad, Iran; 7 Neuroscience Research Center, Mashhad University of Medical Sciences, Mashhad, Iran; 8 Dr. Farhud’s Genetic Clinic, Tehran, Iran; 9 School of Advanced Medical Science, Islamic Azad University, Tehran, Iran; 10 National Institute of Genetic Engineering and Biotechnology, Tehran, Iran; 11 Department of Neurology, Johns Hopkins Medicine, Baltimore, MD USA; 12 Jefferson Institute of Molecular Medicine, Thomas Jefferson University, Philadelphia, PA USA; 13 Department of Dermatology and Cutaneous Biology, Sidney Kimmel Medical College, Thomas Jefferson University, Philadelphia, PA USA; 14 Pediatric Neurology Division, Children’s Medical Center, Pediatrics Center of Excellence, Ataxia Clinic, Tehran University of Medical Sciences, Tehran, Iran

**Keywords:** Neuroaxonal dystrophy, Mutation, *PLA2G6*, PLAN, INAD, *PLA2G6*-associated dystonia–parkinsonism

## Abstract

**Background:**

Phospholipase-associated neurodegeneration (PLAN) caused by mutations in the *PLA2G6* gene is a rare neurodegenerative disorder that presents with four sub-groups. Infantile neuroaxonal dystrophy (INAD) and *PLA2G6*-related dystonia-parkinsonism are the main two subtypes. In this cohort, we reviewed clinical, imaging, and genetic features of 25 adult and pediatric patients harboring variants in the *PLA2G6*.

**Methods:**

An extensive review of the patients’ data was carried out. Infantile Neuroaxonal Dystrophy Rating Scale (INAD-RS) was used for evaluating the severity and progression of INAD patients. Whole-exome sequencing was used to determine the disease's underlying etiology followed by co-segregation analysis using Sanger sequencing. In silico prediction analysis based on the ACMG recommendation was used to assess the pathogenicity of genetic variants. We aimed to survey a genotype-genotype correlation in *PLA2G6* considering all reported disease-causing variants in addition to our patients using the HGMD database and the chi-square statistical approach.

**Results:**

Eighteen cases of INAD and 7 cases of late-onset PLAN were enrolled. Among 18 patients with INAD, gross motor regression was the most common presenting symptom. Considering the INAD-RS total score, the mean rate of progression was 0.58 points per month of symptoms (Standard error 0.22, lower 95% − 1.10, and upper 95% − 0.15). Sixty percent of the maximum potential loss in the INAD-RS had occurred within 60 months of symptom onset in INAD patients. Among seven adult cases of PLAN, hypokinesia, tremor, ataxic gate, and cognitive impairment were the most frequent clinical features. Various brain imaging abnormalities were also observed in 26 imaging series of these patients with cerebellar atrophy being the most common finding in more than 50%. Twenty unique variants in 25 patients with PLAN were detected including nine novel variants. Altogether, 107 distinct disease-causing variants from 87 patient were analyzed to establish a genotype–phenotype correlation. The P value of the chi-square test did not indicate a significant relationship between age of disease onset and the distribution of reported variants on *PLA2G6*.

**Conclusion:**

PLAN presents with a wide spectrum of clinical symptoms from infancy to adulthood. PLAN should be considered in adult patients with parkinsonism or cognition decline. Based on the current knowledge, it is not possible to foresee the age of disease onset based on the identified genotype.

**Supplementary Information:**

The online version contains supplementary material available at 10.1186/s13023-023-02780-9.

## Introduction

Phospholipase-associated neurodegeneration (PLAN) is an extremely rare neurodegenerative disorder characterized by accumulation of extra iron in the basal ganglia, axonal swelling, and presence of the spheroid bodies throughout the peripheral and central nervous systems including cerebellar cortex, globus pallidus, brainstem, and dorsal column of the spinal cord [1–3]. Phospholipase A2 Group VI; *PLA2G6* gene (*PLA2G6;* OMIM: 603604) encodes a calcium-dependent phospholipase A2 (CaI-PLA2) which catalyzes the hydrolysis stage of the ester bonds in fatty acyl groups of phospholipids and produces free fatty acids and lysophospholipids [4, 5]. This gene is mapped on the q arm of chromosome 22 (22q13.1) and contains 17 exons that span more than 69 k base pairs [5].

In addition to the phospholipid metabolism alteration, pathogenic variants of *PLA2G6* can result in failure to repair oxidative damage to phospholipid membranes. These changes are detrimental to membrane fluidity and permeability, and may contribute to the pathology associated with PLAN [1–3]. The high metabolic demand and exposure to oxidative stress in the central nervous system cells, particularly in areas such as the cerebellum and basal ganglia, make these structures highly susceptible to changes in their phospholipase A2 activity in case of abnormal *PLA2G6* [1, 6]. On neuroimaging, cerebellar atrophy with signal hyperintensity in the cerebellar cortex, iron deposition in the globus pallidus and substantia nigra (SN), and calval hypertrophy may be observed [16].

According to the phenotype, age at onset, and rate of disease progression, *PLA2G6*-associated neurodegeneration (PLAN, OMIM: 610217) is usually classified into four subgroups: infantile neuroaxonal dystrophy (INAD, NBIA2A; MIM# 256600), atypical neuroaxonal dystrophy (ANAD, NBIA2B; MIM# 610217), *PLA2G6*-related dystonia-parkinsonism (PARK14; MIM# 612953), and autosomal recessive early onset Parkinsonism (AREP) [4]. Molecular studies confirm the diagnosis of PLAN by finding homozygous or compound heterozygous mutations in the *PLA2G6* gene, while PLAN subtypes are mainly differentiated based on age of onset and clinical manifestations; however, clinical, neurophysiologic, radiographic, and laboratory findings are important in determining the specific phenotype of PLAN [4, 7, 8].

INAD commonly manifests with a progressive difficulty in gross motor milestones achievement in children such as hypotonia, inability to sit or control the head, and gait disturbance. Other important clinical symptoms are visual problems, such as visual disturbances, optic atrophy, and pendular nystagmus. Visual disturbances can affect vision and depth perception, and consequently affect motor coordination and balance secondarily. As the disease progresses, spastic tetraparesis develops with symmetrical pyramidal signs, progressive cognitive decline, optic atrophy, and bulbar dysfunction [6, 9–11]. Despite supportive care, patients rarely survive beyond their first decade. In few cases, patients report seizures early or late in the disease course [6, 10, 12–14]. The initial presentation of ANAD may resemble INAD; however, it is more commonly accompanied by speech delay, autistic features, and emotional instability, as well as ataxia, extrapyramidal symptoms, dysphagia and feeding difficulties. Of note, the clinical progression of ANAD is slower than INAD [4]. Patients with PARK14 and AREP usually develop symptoms in adulthood, and they often have a normal appearance and proper milestones achievement during childhood [4]. Bradykinesia and tremors with dystonia, cognitive regression as well as gait instability are the most prominent clinical features of these patients [4].

Defining the natural history of a PLAN as a rare neurodegenerative disorders is important, because it will help health care professionals to devise timely and appropriate management and follow-up strategies of affected patients as well as determining the disease prognosis. Therefore, more clinical, imaging, and genomic data is needed to get insight into the natural history of the disease. Currently, no clinical measures of disease status or progression of PLAN is available because of its status as an ultra-orphan disease [4]. Herein, we reviewed the clinical, imaging, and genomic findings of twenty-five patients with PLAN, including eighteen cases with clinical aspects of INAD and seven cases with a clinical spectrum of late-onset PLAN (PARK14 and AREP). Using Whole-exome sequencing (WES), we aimed to identify underlying variants in *PLA2G6* and by comparing with all previously reported variants delineate a possible phenotype-genotype correlation in patients harboring *PLA2G6* variants.

## Material and methods

### Ethical approval

Informed consent was obtained from all adult participants as well as the parents of pediatric patients as their legal guardians. The study was approved by ethics committee of Tehran University of Medical Sciences, and the ethics committee of the National Institute for Medical Research Development (NIMAD) of Iran under the codes of ID: IR.NIMAD.REC.1397.508, and ID: IR.NIMAD.REC.1399.066.

### Patients

The present study analyzed comprehensively eighteen genetically confirmed cases of children with INAD and seven cases of late-onset PLAN. All patients underwent neurological evaluations at their first visit. Subsequent follow-up visits were also analyzed if any data was available. An extensive review of the patient's developmental milestone achievement, medical, and family history, age of disease onset, and neurological and ophthalmologic findings was carried out. Whenever needed, patients' families were contacted to clarify information obtained from the medical records. INAD rating scale (INAD-RS) were filled out for pediatric patients at their first visit [15]. Almost all patients underwent T1-, T2-, and T2* weighted magnetic resonance imaging (MRI). All available brain MRI series of patients were reviewed by two neurologists and one neuroradiologist and a consensus was achieved in cases of disagreement.

### The Infantile Neuroaxonal Dystrophy Rating Scale (INAD-RS)

INAD-RS as a validated structured questionnaire was used for evaluating the severity and progression of INAD patients [17]. INAD-RS is a combination of history and physical examination questions that assess the patients’ (1) ability to carry on their daily life activities and functions, (2) ability to interact with the environment and their caregivers, and (3) symptomatic status related to how they feel. The INAD-RS is a 40-items scale (each item scoring from 0 to 2 points) consisting of 6 categories: (1) gross motor-and-truncal-stability function (24 total scores); (2) fine motor skills (12 total scores); (3) bulbar function (14 total scores); (4) ocular examination (10 total scores); (5) temporo-frontal function (16 total scores); and (6) autonomic nervous system (4 total scores). The total INAD-RS score is the sum of these 6 groups’ scores, which ranges from 0 to 80, lower scores representing the higher severity of the disease.

### Whole-exome sequencing and family segregation

The salting-out standard procedure was used to extract genomic DNA from whole blood samples of the probands. Whole-exome sequencing was used to determine the disease's underlying etiology, and after detecting variants, co-segregation analysis was examined using Sanger sequencing.

### Prediction of pathogenicity and population database

In silico prediction analysis based on the ACMG recommendation was used to assess the pathogenicity of genetic variants. To predict the pathogenicity of genetic variants on gene function in silico, we used Clinvar (https://www.ncbi.nlm.nih.gov/clinvar/), Varsome (https://varsome.com/), Franklin (https://franklin.genoox.com/clinical-db/home) databases, and InterVar software (http://wintervar.wglab.org/). Each mutation was assigned as 'Benign' (class 1), 'Likely benign' (class 2), 'Uncertain significance' (class 3), 'Likely pathogenic' (class 4), or 'Pathogenic' (class 5). We applied four in silico algorithms to estimate the impact of gene variants on function: Mutation Taster (http://www.mutationtaster.org), SIFT (http://sift-dna.org), FATHMM-MKL (https://fathmm.biocompute.org.uk/fathmmMKL.htm). In two databases, including gnomAD and Iranome, we checked the distribution of minor allele frequencies (MAFs) of these variants.

For genotype–phenotype correlation, we used the Human Genome Mutation Database (HGMD) professional database. This database assigns some special tags for each variant, which are DM for disease-causing mutation, DM for likely disease, DP for disease-causing mutation associated polymorphism, FP for in vitro or in vivo functional polymorphism, DFP for disease-category associated polymorphism with additional functional evidence, and R for retired record. The DM, or DM? variants were selected for our study, and among these variants, we included those variants that were pathogenic and likely-pathogenic in each of Franklin or ClinVar databases. We excluded big deletions and duplications and evaluated all 17 exons of the *PLA2G6* gene focusing on those exons who have been reported harboring disease-causing variant to find a probable genotype–phenotype correlation with the two main clinical forms of PLAN, INAD versus adult-onset. Based on the age of disease onset, we examined all reported variants at HGMD professional database in addition our variants-in each of the 17 exons of the *PLA2G6* gene and assessed them using the chi-square statistical approach.

### Structures and alignments

We employed a prediction approach with the AlphaFold to examine the protein structure (https://alphafold.ebi.ac.uk/entry/O60733). We used the UCSC databases (https://genome.ucsc.edu) and ConSurf (https://consurf.tau.ac.il/) to carry out the analysis of protein conservation across species. According to Additional file 1, "Clustal Omega was used to carry out sequence alignments of the human PLA2G6 protein. The results for the sequences sp|O60733 (HUMAN) and sp|E1BB89 (BOVIN) and sp|P97819 (MOUSE) are pairwise alignments with regard to those two other sequences. The Clustal Omega program, which may be found online at https://www.ebi.ac.uk/Tools/msa/clustalo/, was used to create the alignments. ScanProsite was used to examine the protein families and domains. According to this database the ankyrin repeat profile (PS50088), the ankyrin repeat region circular profile PS50297, and the patatin-like phospholipase domain (ps51635) are three conserved domains or motifs found in the PLA2G6 protein sequence (https://prosite.expasy.org/scanprosite/). In addition, PyMOL V2.4.1 (https://pymol.org), I-mutant V2.0 (https://folding.biofold.org/cgi-bin/i-mutant2.0.cgi), and DynaMut (http://biosig.unimelb.edu.au/dynamut/) were used to investigate the effects of variants on protein structure and protein flexibility and stability. We demonstrated the precise residue-by-residue stereochemical quality using the RAMPAGE online tool and the Ramachandran plot. (https://zlab.umassmed.edu/bu/rama/). The probable genetically intolerant locations and regions that might have affected the function of the protein were also visualized using MetaDome. PyMOL V2.4.1 (https://pymol.org) software displayed the three-dimensional structure of the protein and the interaction of amino acids before and after the novel variants, and the I-TESSER database displayed for the frameshift variant (c.869_870insAGCCC) and modeled the three-dimensional structure of the protein after the frameshift variant.

## Results

### Clinical findings of INAD cases

Eighteen children from eighteen individual families including thirteen males and five females with molecular diagnosis of PLAN were enrolled in this study with a range of 19–130 months since symptom onset (mean of 62.6 months, SD: 30.5). The range of disease onset age was 0–108 months (mean of 22.4, SD: 24.6). Table 1 summarizes the demographic and clinical characteristics of patients. It includes mainly their age at disease onset (months), the initial presenting symptoms, and the status of other important reported neurologic symptoms in INAD such as cognitive impairment, seizure, and hearing impairment during the disease course in our patients. Besides, the results of performed diagnostic tests consisting of electromyography-nerve conduction velocity study (EMG-NCV), and brain MRI findings have also been demonstrated. Gross motor regression was the most common initial presenting symptom (55.55%) among our patients. Cognitive impairment, seizures, and hearing impairment were all reported in 61.11%, 27.77%, and 27.77% of patients during the disease course, respectively. Moreover, some other cardinal neurologic symptoms such as visual disturbance, and bulbar dysfunction were noted in 77.77%, and 77.77%, respectively.

**Table 1:** Demographic and clinical characteristics of INAD patients

Case	Sex	Consanguinity	Pregnancy history	Family history	Age at disease onset (M)	Age at the time of study (M)	First presentation symptoms	Cognitive impairment	Seizure	Hearing impairment	Visual disturbance	Bulbar dysfunction	EMG-NCV	Brain MRI
1	M	First cousin	N	N	12	60	MDD	No	No	No	Yes	No	Chronic axonal type motor neuropathy	Cerebellar hemispheres and vermis atrophy
2	M	First cousin	N	N	18	90	MR	No	No	Yes	Yes	Yes	Chronic axonal type motor neuropathy	Cerebellar hemispheres and vermis atrophy, supra and infra tentorial atrophy, corpus callosum atrophy, widening of Sylvian fissure, vertical splenium of corpus callosum, clavarial hypertrophy
3	M	First cousin	N	N	108	141	MR	No	No	No	No	No	NL	Cerebellar atrophy
4	F	Remotely consanguineous	N	N	15	61	MR	Yes	No	No	Yes	Yes	Chronic axonal type motor neuropathy	Vermis atrophy, cortex atrophy, Posterior corpus callosum dysplasia, iron accumulation in Globus pallidus
5	M	Remotely consanguineous	N	N	13	56	MR	No	No	No	Yes	Yes	Chronic axonal type motor neuropathy)	Cerebellar atrophy, hypomyelination, clavarial hypertrophy
6	M	First cousin	N	N	5	85/E	Nys	No	No	Yes	Yes	Yes	Chronic axonal type motor neuropathy	Cerebellar vermis and hemispheres atrophy, vertical splenium of corpus callosum
7	M	First cousin	N	Brother	60	99	AG	No	No	No	No	No	NA	Cerebellar atrophy, mild inferior vermian hypoplasia, mega cisterna magna
8	M	First cousin	N	N	At birth	48	DD	Yes	No	No	Yes	Yes	Chronic axonal motor polyneuropathy	Cerebellar atrophy and calvarial hypertrophy
9	M	First cousin	N	Uncle	12	31	MDD	Yes	No	No	No	No	NA	Cerebellar atrophy
10	F	N	N	Sister	24	154	MR	Yes	No	No	Yes	Yes	NA	NA
11	M	First cousin	N	N	16	99	MR	Yes	Yes	Yes	Yes	Yes	Chronic axonal type distal sensorimotor polyneuropathy	Cerebellar atrophy, global brain atrophy, Hypomyelination
12	F	First cousin	N	N	22	48	MR	Yes	Yes	No	No	Yes	Chronic axonal type motor neuropathy	NL
13	M	First cousin	N	N	7	93	MDD	Yes	No	Yes	Yes	Yes	Chronic axonal type motor neuropathy	Cerebellar atrophy, Basal ganglia iron deposition on T2
14	M	First cousin	N	Cousin	12	43	MDD	Yes	Yes	No	Yes	Yes	Chronic axonal type motor neuropathy	Hypoplasia of inferior vermis & cerebellar atrophy
15	F	First cousin	N	N	18	112/E	Nys	Yes	No	No	Yes	Yes	NA	Cerebellar vermis and hemispheres atrophy
16	M	Remotely consanguine	N	N	24	96/E	MR	Yes	Yes	No	Yes	Yes	NA	Hypoplasia of inferior vermis, cerebellar atrophy, enlargement of fourth ventricle, global cerebral atrophy
17	M	First cousin	N	N	20	89/E	MR	No	No	No	Yes	Yes	Chronic severe axonal type sensory-motor polyneuropathy	Cerebellar atrophy
18	F	Second cousin	N	N	18	126	MR	Yes	Yes	Yes	Yes	Yes	NA	Dilated ventricles and extra axial CSF spaces representing significant volume loss and cerebral atrophy, Thinness of Corpus callosum, Optic nerve atrophy

*M* male, *F* female, *N* none, *MDD* motor developmental delay, *MR* motor regression, *Nys* nystagmus, *AG* ataxia gait, *DD* developmental delay, *NA* not available, *NL* normal, *CSF* cerebrospinal fluid

**Fig. 1:**
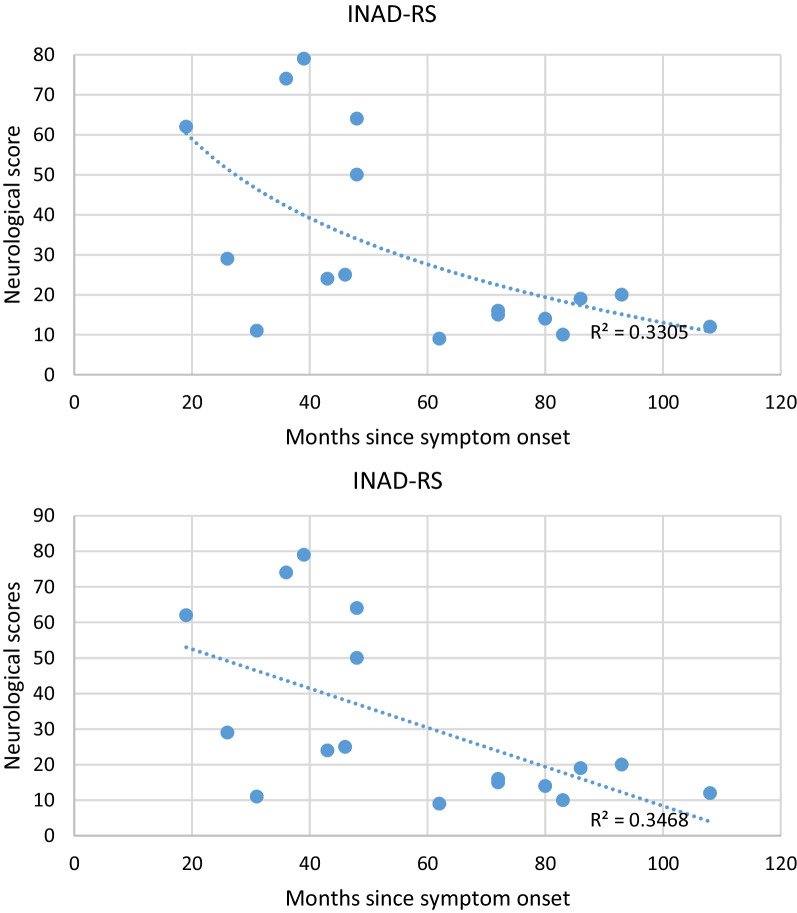
INAD-RS Progression. Figure showing months since symptom onset and neurological score on X-axis and Y-axis, respectively. There was a negative correlation between months since symptom onset and total score of INAD-RS. An average loss of 0.58 points per month of symptoms was observed. The best fit trend line was linear (r^2^ = 0.34)

The INAD-RS total score ranges at the time of enrollment in the study were 9–74 (mean: 58.35, SD: 25.87, and median: 22). Based on the INAD-RS scores, the medians of the gross motor, fine motor, bulbar, ocular, temporo-frontal and autonomic functions were 0, 0, 10, 6, 2.5, and 4, respectively (Table 2). In general, following the initial motor symptoms in INAD patients, the disease progressed steadily, leading to finally ocular, temporo-frontal, bulbar, and autonomic manifestations. After excluding one patient (case number: 10) due to the incompatibility of phenotype severity and the time in months elapsed since the symptom onset, the rate of progression on INAD-RS was determined using a linear regression model to cross-sectional data from 17 participants (Fig. 1). The mean rate of progression was 0.58 points per month of symptoms (Standard error 0.22, lower 95% − 1.10, and upper 95% − 0.15). The best-fit trend lines were linear (r^2^ = 0.34), and logarithmic (r^2^ = 0.33), respectively. The reason for a logarithmic slope closely associated with progression appears to be due to the increased loss of function mostly during the initial stages of the disease. Indeed, once most parts of the milestones regression happen at the beginning of the disease, the residual function has less variance to detect any differences across the remaining disease course. Sixty percent of the maximum potential loss in the INAD-RS had occurred within 60 months of symptom onset. This provides an optimum window for potential therapeutic intervention in INAD patients. We discovered a statistically significant correlation between the scale score and months since symptom onset. However, this correlation was low in mangnitude (r^2^ = 0.33, *p* = 0.013). Moreover, it should be noted that Fig. 1 shows quite a large variability between patients, and 2 separate clinical groups with potentially different clinical decline over time might be distinguished, those with a very low score prior to 60 months since symptom onset and those with a still relatively high score after 60 months since symptom onset.

**Table 2:** The infantile neuroaxonal dystrophy rating scale (INAD-RS) of INAD patients

Case	Gross motor (0–24)	Fine motor (0–12)	Bulbar function (0–14)	Ocular (0–10)	Temporo-frontal (0–16)	Autonomic (0–4)	Total (0–80)
1	22	12	14	7	5	4	64
2	1	0	6	4	1	4	16
3	21	12	14	10	14	3	74
4	0	0	10	7	4	4	25
5	0	0	11	7	2	4	24
6	0	0	10	0	1	3	14
7	23	12	14	10	16	4	79
8	12	7	11	6	10	4	50
9	14	9	14	10	11	4	62
10	14	12	12	8	16	4	66
11	0	0	2	4	1	3	10
12	0	0	12	10	3	4	29
13	0	0	10	4	1	4	19
14	0	0	8	0	0	3	11
15	0	0	6	0	1	2	9
16	0	0	9	2	1	3	15
17	0	0	9	6	3	2	20
18	0	0	9	0	1	2	12

**Fig. 2:**
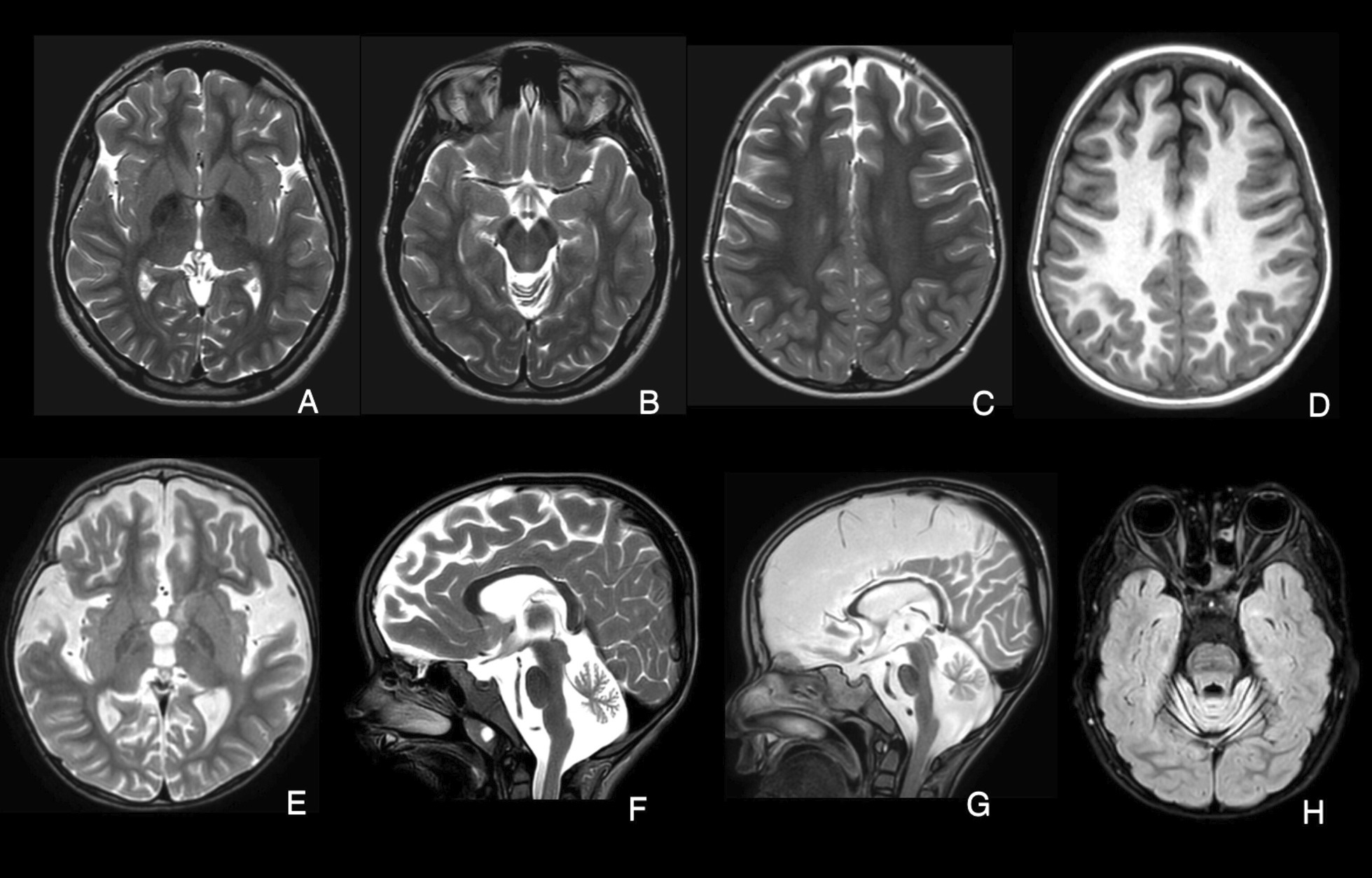
MRI-based imaging findings in patients. A-B) Patient with PLA2G6 due to c.2370 T > G and c.1516G > A variants; Axial T2-weighted images indicate hypointensity of the Globus pallidus (**a**) and substantia nigra (**b**) denoting iron deposition at the age of 11 years. **c, d** Patient with PLA2G6 due to c.668C > T variant; Axial T2 & T1-weighted images show white matter hypomyelination at the age of 4 years. **e, f** Patients with PLA2G6 due to c.668C > T variant and c.668C > T variant; Axial and sagittal T2-weighted images show cerebral atrophy and cerebellar atrophy at the age of 4 years. **g** Patient with PLA2G6 due to c.668C > T variant; Thinning of corpus callosum and calval hypertrophy at the age of 4 years. **h** Patient with PLA2G6 due to c.668C > T variant; The initial stage of optic atrophy on axial FLAIR sequence at the age of 4 years

Twenty brain MRIs were analyzed for seventeen pediatric patients which revealed cerebellar atrophy as a universal feature observed in all except one patient (case number: 3). We also observed some other features in INAD patients including widening of the cerebral sulci and thinning of corpus callosum, iron accumulation in globus pallidus, and optic nerve atrophy. In two cases, we observed cerebral hypomyelination (Fig. 2). Of note, these MRI series were performed at different time points of the disease course.

### Clinical findings of adults cases

A total of seven adult cases of PLAN from five individual families harboring a variant in *PLA2G6* were enrolled. The mean age of the patients was 25 ± 7 years (age range, 17–35 years) including three males (43%) and four females (57%). All cases were from consanguineous parents and four of them were two by two siblings. Details of patients’ demographic, clinical and Para clinical characteristics have been presented in Table 3.

**Table 3:** Clinical and para clinical findings of adult patients

Case	Sex	Consanguinity	Family history	Age at disease onset (Y)	Age at the time of study (Y)	First presentation symptoms	Cognitive impairment	Seizure	Others	EMG-NCV	Brain MRI
1	M	First cousin	Similar problem in his sister	16	18	HT, HK	Yes	No	PK, PR, IT	NL	Vertically oriented corpus callosum
2	F	First cousin	Similar problem in her brother	13	17	HT, HK	No	No	PK, IT	NL	NA
3	M	First cousin	Similar problem in his sister	12	29	DI, GD	No	No	PK, DA	Sensory motor polyneuropathy	Cerebellar and cervical cord atrophy
4	F	First cousin	Similar problem in her brother	7	32	MS, GD	Yes	No	No	NA	Cerebellar atrophy, nonspecific white matter hyper intensity on T2 and FLAIR in centrum semiovale
5	F	First cousin	No	18	23	HT, HK, BK	No	No	PK, SP, HR, BB	NA	Mild cerebellar atrophy
6	M	First cousin	Down syndrome in brother	16	21	MS, DA, CD	Yes	Yes	AG, PK, HR, AP	NA	Hypo intensity of Globus pallidus and substantia nigra on FLAIR, T2 and SWI sequences. Severe diffuse brain atrophy and brain stem (more prominent in frontotemporal areas), Iron deposition in Globus pallidus and SN
7	F	Distant relative	No	25	35	MS	No	No	AR-PK, GRL	NA	Mild cerebellar atrophy

*M* male, *F* female, *N* none, *AG* ataxia gait, *DD* developmental delay, *NA* not available, *NL* normal, *CSF* cerebrospinal fluid, *HT* hand tremor, *HK* hyperkinesia, *BK* bradykinesia, *PK* parkinsonism, *PR* psychomotor retardation, *IT* intension tremor, *GD* gait disturbance, *DI* disequilibrium, *DA* dysarthria, *MS* movement slowness, *SP* spasticity, *HR* hyperreflexia, *BB* babinski, *CD* cognitive decline, *AP* apraxia, *AR-PK* akinetic-rigid Parkinsonism, *GRL* good response to levodopa

The mean age of the disease onset among adult cases was 15.3 ± 5.5 years and the most common initial presenting symptoms were hypokinesia (6/7), and hand tremor (3/7). Ataxic gate (1/7), hyperreflexia(2/7), dysarthria (2/7), and cognition decline (1/7) were other symptoms which were seen on examinations. History of seizure was reported in only one case. Six MRI series were available to analyze for these 7 adult patients. The most common findings was cerebellar atrophy (4/7, 57%). Vertically oriented corpus callosum and iron deposition in globus pallidus and substantia nigra were other imaging findings, each were observed in one patient. Like INAD patients, these MRI series were prformwd at different time points of disease course. EMG-NCV study was performed for three of these cases which was normal in two of them and findings of third patient was compatible with axonal sensory motor polyneuropathy.

### Molecular findings

#### Pathogenicity prediction of variants

All 17 exons and exon–intron boundaries of *PLA2G6* were documented according to the current mutation nomenclature guidelines of the Human Genome Variation Society based on the canonical transcript with the GenBank accession number NM_003560.4. In current study, twenty unique variants in 25 patients with PLAN disease were detected, including 15 missense variants (75%), 2 nonsense (10%), 1 frameshift (5%), and 2 splicing site variants (10%), implying that most of the variants were missense type. Nine novel variants, including a frameshift (c.869_870insAGCCC) and 8 missense variants were included in these 20 detected variants. Pathogenicity classification of all variants was done according to ACMG guidelines, which resulted in 10 likely-pathogenic (50%), 8 pathogenic (40%), and 2 VUS variants (10%). In all variants, post transcription effects were assessed using the SIFT database and showed “Damaging” for all except for c.1774C > A which was “tolerant” and c.1862C > A which had “Uncertain Significance”.

Mutation Taster also showed “Disease-Causing” for all detected variants, except for the c.1516G > A variant which was a “Polymorphism” and the c.1862C > A variant which had “Uncertain Significance” based on this database. Besides, all the variants according to the FATHMM-MKL database were “Damaging”. In this study, we had seven patients with adult-onset type of PLAN. Four cases had the c.1862C > A variant in common, two of them shared the c.1715C > T variant, and one of the patients showed the c.1946G > A. All patients who had the missense variants were homozygous. Moreover, we had 18 patients with INAD, two were compound heterozygous. One of them had the c.962 T > C and c.1039G > A variants, both of these missense variants were on exon 7. The other one had a nonsense variant of c.2370 T > G and missense variant of c.1516G > A which were on exons 17 and 11, respectively. The remaining of the patients with INAD had a homozygous variant.

#### Population databases and structural prediction

**Fig. 3:**
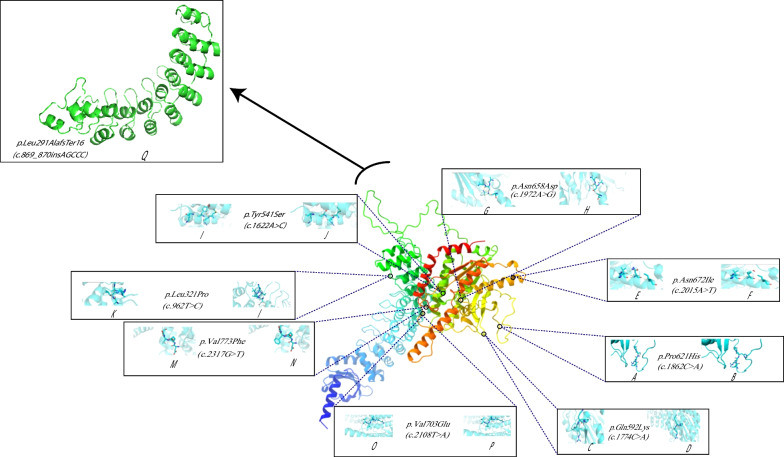
The nine novel variants in our study have been shown. We displayed the positions of the variant-type residues (**b, d, f, h, j, l, m, p**) and the wild-type residues (**a, c, e, g, i, k, m, o**) on the protein. With the exception of one variant, a frameshift variant, all of other variants were missense. We used the I-Tesser database to anticipate how this mutation would affect the protein's structure

We performed an analysis on the database of gnomAD populations. Only two out of our twenty unique variants were present in the gnomAD population database: c.2370 T > G in exon17 and c.1903C > T in exon14. The frequency of these variants was 0.0059 percent and 0.0007 percent, respectively. Other variants were not included in this population database. In addition, none of the variants were discovered in the Iranom database. Most of the identified variants were highly conserved, according to the ConSurf and UCSC database. Besides, most of the variants we discovered in patients can reduce the stability of the PLA2G6 according to I-mutant V2.0, DynaMut (http://biosig.unimelb.edu.au/dynamut/), and PyMOL V2.4.1 (https://pymol.org). The structure modeling of PLA2G6 protein using RAMPAGE online tool indicated that almost 98.868 percent of residues are in the most favored regions, around 0.707 percent of residues are in the allowed regions, and only 0.424 percent of residues are in the outlier regions, which suggested that the predicted structure of AF-O60733-F1-model_v2.pdb was acceptable (Fig. 3). The effect of the frameshift variant (NM_003560.4:c.869_870insAGCCC) on protein structure using I-TESSER database has also been shown in Fig. 3 with a C-score of − 0.43 and estimated TM-score and RMSD of 0.66 ± 0.13 and 7.2 ± 4.2 Å, respectively.

#### Genotype–phenotype correlation in *PLA2G6*

**Fig. 4:**
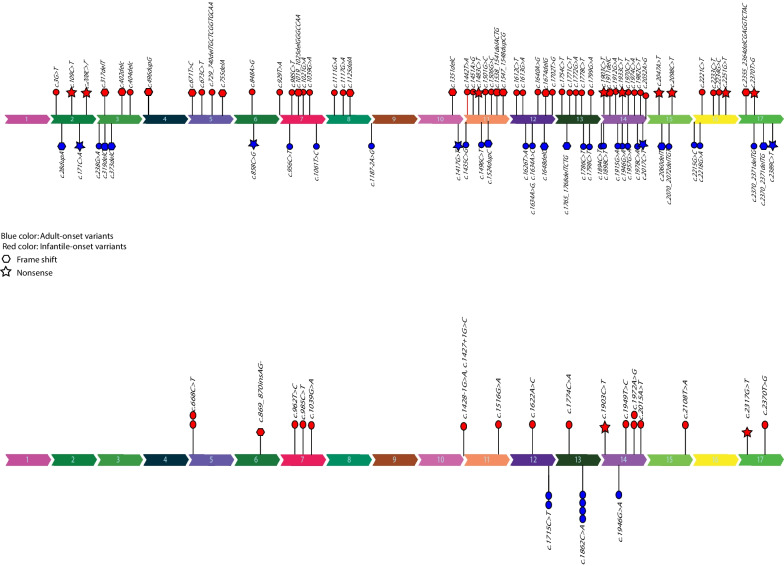
The distribution of reported *PLA2G6* variants and their pathogenicity evaluation. **a** The HGMD database variants for patients with early-onset disease (red) and patients with adult-onset disease (blue) are shown, separately in this section. Exons 1 and 9 did not have variants in any of the disease types, while exons 4 and 5 did not have variants in INAD patients. The frequency of variants seems to be higher in the gene's terminal regions. **b** This section shows the variants were analyzed in this study separately for patients with early onset (red color) and patients with late-onset (blue color). The variants related to patients with adult-onset were found on exons 12, 13, and 14, while the variants associated with INAD seem to be dispersed throughout the gene.

In order to determine a genotype–phenotype correlation, we evaluated the pathogenic and likely pathogenic variants reported in HGMD for *PLA2G6* gene depending on the age of disease-onset. We collected a total of 107 distinct *PLA2G6* variants from 87 patients whose mutations were cataloged in the Human Gene Mutation Database including our patients. We discovered that 71 variants were linked to INAD patients, whereas 36 variants were linked to patients with adult-onset. Overall, we analyzed 52 pathogenic variants, 53 likely pathogenic variants, and 2 variants of unknown significance (VUS). The distribution of reported variants seemed to be higher near the gene's terminal regions. The variants related to patients with adult-onset were inside exons 12, 13, and 14, while the variants associated with INAD seem to be dispersed throughout the gene. We observed that in adult-onset patients no variant in exons 4 and 5 have been reported so far. In addition, no variants have been reported in exons 1 and 9 of this gene in all types of PLAN (Fig. 4a, b).

**Fig. 5:**
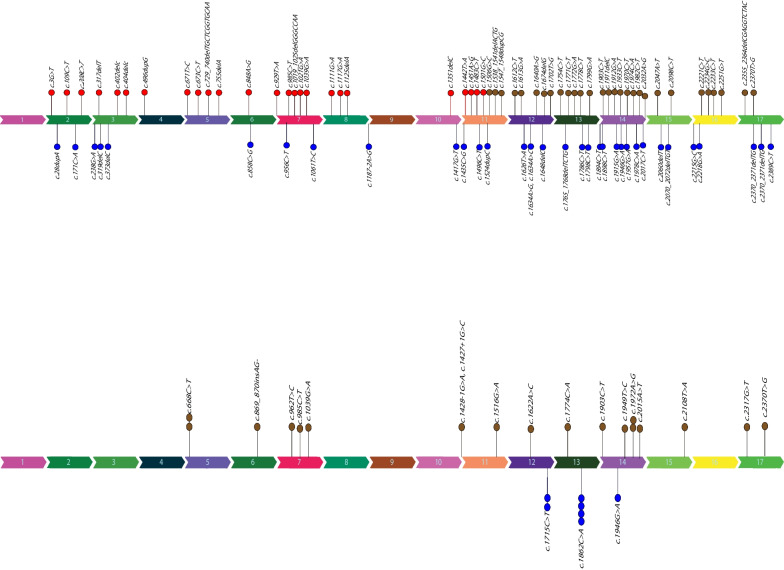
The presentation of variant types (frameshift or nonsense) in two groups of adult-onset and INAD patients. The hexagon represents a frameshift variant, whereas the asterisk represents a nonsense variant. Regarding the variants of HGMD database in patients with PLAN, nine were nonsense and thirteen were frameshift variants. Five of the investigated variants in adult-onset patients were nonsense, and eight were frameshift. Regarding the variants associated with this study, we identified 2 nonsense and 1 frameshift variants in our INAD patients

Otherwise in both forms of the disease, variants are distributed randomly on all exons, and no other obvious association were noticed. We utilized the chi-square statistical tool to quantify this association. We included frameshift and nonsense mutations in our calculation as well. However, we exclude missense variants from the analysis. Based on the variant type, there were 11 nonsense and 14 frameshift variants in patients with INAD, respectively. In addition, 5 variants in adult-onset patients were nonsense, whereas 8 were frameshift. Two of the nonsense and one of the frameshift variants mentioned above were detected in our INAD patients (Fig. 5). The P value of this test did not indicate a significant relationship between distribution of the variants and these two types of disease.

## Discussion

Here, we presented a cohort of 25 patients of pediatric and adults PLAN. The mean age of disease onset in INAD cases was 22.4 months while the mean age at symptoms onset in Altuame et.al. study on 28 INAD patients was 15 months [16]. In another study reporting 10 patients with INAD, the earliest symptoms presented between 6 and 18 months old which is consistent with the age of disease onset in our study [13].

Corroborated with recent cohort studies of INAD patients, the most common initial symptoms in our patients were related to motor function including delays in balance or walking and/or motor milestone regression [15, 16]. However, two of our INAD cases presented with nystagmus. Although nystagmus is a common neurological sign in INAD, it is often seen in the later stages of the disease [9]. Only a case report paper reported downbeat nystagmus in a 13-month-old female as the only presenting symptom. The authors indicated that downbeat nystagmus can be a rare but very early onset sign of cerebellar involvement in INAD and can anticipate the appearance of psychomotor regression and radiological abnormalities [17]. Vermis atrophy was found in both of our cases and also in the Frattini case report, which may be a radiological sign that is correlated with the occurrence of nystagmus in these patients. However, this may not be a pathognomonic finding for the nystagmus in brain MRI as a few other patients reported vermis atrophy without presenting nystagmus. In total, following the initial symptoms in our patients with INAD, the disease progressed steadily, leading to a progressive decline of gross motor milestones followed by fine motor regression and finally appearing of ocular, temporo-frontal, bulbar, and autonomic dysfunctions. This pattern of disease progression is consistent with previous studies [6, 9–11, 13, 14, 16, 18].

Based on a literature review, seizures may develop early or late during the disease course of INAD; however, it has been reported to occur in a minority of patients [6, 10, 12–14]. In a recent cohort study of 28 INAD patients, almost half of the patients had at least one episode of mostly generalized seizure at an average age of 39 months [16]. Although seizure was less common (27.77%) in our study compared to this recent cohort study, it seems reasonable that evaluation of seizure using electroencephalogram (EEG) or video EEG monitoring to be considered in symptomatic treatment and management of INAD patients.

Hearing impairment was also detected in 27.77% of patients in our study using abnormal brain stem auditory evoked response (BAER). In a recent cohort study on INAD patients conducted by Purushothaman et.al., abnormal hearing test results were identified in 10 out of 13 children (77%) [19]. This difference might be caused by more sensitive audiological evaluations including pure-tone audiometry, tympanometry, BAER, and distortion product otoacoustic emissions that were performed in the Purushothaman et.al. study. Autonomic dysfunction in INAD can present with constipation or urinary incontinence [20]. Although urinary inconstancy was detected in none of our patients; eight cases were reported to have constipation requiring treatment. This result is in concordance with the result of a previous study indicating that nine out of twenty-eight INAD patients underwent treatment due to symptomatic constipation [16]. EMG-NCV findings in INAD patients were mostly consistent with the previous studies, as 16 patients showed axonal-type motor neuropathy. Interestingly, two INAD patients showed axonal-type sensorimotor polyneuropathy that has not been reported as commonly as axonal-type motor neuropathy [9, 21]. Altoghether, 4 patients of this cohort showed axonal-type sensorimotor polyneuropathy that could be an interesting finding.

Brain MRI in seventeen pediatric patients revealed cerebellar atrophy as a consistent feature observed in all except one patient. Although cerebellar and vermis atrophy is a key feature of INAD, in line with previous observations, it may be completely absent at the time of disease onset [10, 12, 13]. Therefore, follow-up imaging studies particularly at a later disease stage may be more informative. Other MRI findings that we observed in some patients included diffuse cerebral atrophy, widening of cerebral sulci and thinning of the corpus callosum, and globus pallidus hypointense lesions. These findings have been occasionally described in previously published INAD cases [10, 11, 22, 23]. However, to the best of our knowledge, cerebral hypomyelination has not been noticed in previous INAD cohort studies, while we presented two case with this feature for the first time. The underlying mechanism has been hypothesized in some previous animal studies [24–26]. However, there are some inconsistent findings on whether the myelin loss among the animals with neuroaxonal dystrophy was explained as either a primary phenomenon or secondary to axonal swelling and dystrophy [25, 26].

To the best of our knowledge, this is the first cohort study in which, the disease severity of a part of enrolled patients has been evaluated with INAD-RS since it was represented by Atwal et.al., in 2020 [17]. The INAD-RS total score ranges were 9–74 (mean of 58.35, SD: 25.87) in our study. This result was completely consistent with the INAD-RS score ranges that were reported by Atwal et.al., (INAD-RS score ranges: 7–69) [15]. There was a negative correlation between months since symptom onset and the total score of INAD-RS. An average loss of 0.58 points per month of symptoms in our INAD patients was observed, while it was an average loss of 0.49 points per month of symptoms based on the study that developed and validated the INAD-RS. Moreover, in line with Atwal et.al. study, a wide variety of neurological function decline was observed among INAD patients. This variability might rise from the different genotypes and some other modifiers which should be more investigated in future studies. [15]. In our study, sixty percent of the maximum potential loss in the INAD-RS occurred within sixty months of symptoms onset. This provides an optimum window for potential therapeutic intervention in INAD patients. The months since symptom onset were determined to be the most sensitive indicator of disease progression, more sensitive than absolute age at diagnosis because of the frequent delay in diagnosis. Our study confirms that the rare nature of INAD makes the diagnosis difficult, and affects families frequently on an extended diagnostic odyssey. This delay between symptom onset and diagnosis is consistent with other rare neurogenetic disorders in children [7, 27–30].

In this study we also reported clinical, imaging and molecular findings of seven adult PLAN cases. First symptoms of Juvenile-adult-onset PLAN have reported to be commenced at the age of 18 to 26 years old which is slightly higher compared to our patients with a mean presentation age of approximately 15 years old [31, 32]. Bradykinesia and hypokinesia are well documented initial manifestations of adult-onset PLAN which was found in 6 out of our 7 patients [4, 32]. In line with our findings, a literature review by Karkheiran et. al., showed that of 23 patients with.young- or adult-onset PLAN-dystonia–parkinsonism (PLAN-DP), all cases had at least one of the parkinsonism-related symptoms [33]. At rest tremor and dystonia were the most frequent observed symptoms in more than 70% of reported adult cases in neurologic examinations [33].

Several studies have also frequently reported psychiatric manifestations i.e., cognition impairment, personality disorders, memory impairment, and behavioral changes as a common group of presenting symptoms in adult patients [4, 32–35]. In our study, cognition impairment was observed in more than 40% of our adult patients but other neuropsychiatric symptoms were not evaluated. In previous studies, a few patients have been described with depression and psychosis as the presenting symptoms [36–38]. In a few cases, neuropsychiatric disorders such as dementia, conduct disorder, and cognitive disability have also been reported [33]. These findings highlight the importance of psychiatric assessment in these patients.

Various pathologic ophthalmologic findings have been reported in patients with PLAN-DP [31]. Supranuclear gaze palsy, lid opening apraxia, oculogyric crisis, saccadic pursuit, fragmented saccades, jerky saccadic, and nystagmus were among reported oculomotor abnormalities in PLAN-DP cases [33]. This wide range of visual and eye movement disturbances simplifies the significance of ophthalmic examination in these patients.

Regarding neuroimaging, cerebellar atrophy is a common findings in brain MRI of PLAN-DP cases like INAD patients [39]. However, unremarkable brain imaging is also a frequently reported pattern which is less common in infantile patients. Iron deposition in basal ganglia which is a characteristic feature in INAD patients only has been observed in one third of adult cases [33, 39]. Different abnormalities of corpus callosum such as thinning, vertical orientation, and elongation which are common findings in INAD have also been reported in a few cases of PLAN-DP [39]. Cerebellar atrophy was the most common finding in our cases (4/6 MRIs), however; none of the patients had a normal brain imaging.

Only a small number of studies describe the relationship between the genotype and phenotype in patients with *PLA2G6* mutations. According to Laura A. and et al. in 2010, the full loss of enzyme function brought on by the V691del mutation raises the possibility that there is a connection between the severity of *PLA2G6* function impairment and the early onset phenotype. According to Yu-Pei Guo et al.'s 2016 study, patients with PLAN may exhibit a range of symptoms and physiological changes as a result of distinct mutations discovered in the *PLA2G6* gene. However, the precise mechanism by which the mutations result in these various outcomes is still unknown.and additional studies might be needed to categorize the mutations based on the 3D structure of the iPLA2β protein. Another study by Brent Bluett et al. in 2019 reports patients with null mutations exhibited with clinical symptoms early in life and acquired more severe clinical phenotypes. Overall, it appears that other factors, such as environmental or genetic modifiers, may also play significant roles in influencing the variability of the disease presentation, even though some studies suggest possible correlations between particular mutations and specific phenotypic presentations (https://doi.org/10.1371/journal.pone.0076831). We checked all published papers of NAD on the HGMD database (www.hgmd.cf.ac.uk/ac/index.php). We found that most reported variants in *PLA2G6* overlap between the two types of PLAN. We discovered that some variants were only seen in the INAD, whereas others were only detected in PLAN-DP, the late onset form of the disease. Accordingly, we assumed that some variants predispose patients to INAD type whereas a few other variants predispose patients to late onset disease. However, we utilized the chi-square statistical tool to quantify the phenotype-genotype correlation in PLAN, but the P value of this test did not indicate a significant relationship between the genotype and age of disease onset.

## Conclusion

PLAN presents with a wide spectrum of clinical symptoms from infancy to adulthood. It should be considered in adult patients with parkinsonism or cognition decline as well as in infants with early-onset hypotonia, neurodegeneration, and cerebellar atrophy. We reported the phenotype and genotype spectrum of a cohort of 25 patients with PLAN. Gross motor regression was the most common presenting symptom in INAD while hypokinesia, tremor, ataxic gate, and cognitive impairment were the most frequent clinical features in adult cases. Cerebellar atrophy was the most common imaging finding in more than half of patients. The P value of the chi-square test didn’t confirm a significant relationship between age of disease onset and the distribution of all reported *PLA2G6* variants on HGMD database in PLAN. Therefore, based on the current knowledge, it is not possible to foresee the age of disease onset based on the identified genotype.

## Supplementary Information

**Additional file 1**. Results of Clustal omega on sequence alignment of the human PLA2G6 protein.

## Data Availability

Data are available by request.

## Abbreviations

ACMG: American College of Medical Genetics and Genomics
AREP: Autosomal recessive early onset Parkinsonism
BEAR: Brain stem auditory evoked response
EEG: Electroencephalogram
EMG-NCV: Electromyography-Nerve conduction velocity study
HGMD: Human Genome Mutation Database
INAD: Infantile neuroaxonal dystrophy
INAD-RS: INAD rating scale
MAFs: Minor allele frequencies
MRI: Magnetic resonance imaging
NAD: Neuroaxonal dystrophy
PARK: *PLA2G6*-Related dystonia-parkinsonism
PLA2G6: Phospholipase A2 Group VI
PLAN: *PLA2G6*-Associated neurodegeneration
RAMPAGE Tool: RNA Annotation and Mapping of Promoters for the Analysis of Gene Expression tool
SIFT: Sorting Intolerant from Tolerant
SN: Substantia nigra
UCSC Database: University of California Santa Cruz database
VUS: Variant of unknown significance
WES: Whole-exome sequencing
