# Correction: Phenotype and genotype heterogeneity of PLA2G6-associated neurodegeneration in a cohort of pediatric and adult patients

**DOI:** 10.1186/s13023-023-02794-3

**Published:** 2023-07-17

**Authors:** Ali Zare Dehnavi, Maryam Bemanalizadeh, Seyyed Mohammad Kahani, Mahmoud Reza Ashrafi, Mohammad Rohani, Mehran Beiraghi Toosi, Morteza Heidari, Sareh Hosseinpour, Behnam Amini, Shaghayegh Zokaei, Zahra Rezaei, Hajar Aryan, Man Amanat, Hassan Vahidnezhad, Pouria Mohammadi, Masoud Garshasbi, Ali Reza Tavasoli

**Affiliations:** 1 Department of Pediatrics, Division of Pediatric Neurology, Children’s Medical Center, Pediatrics Center of Excellence, Tehran University of Medical Sciences, Tehran, Iran; 2 Child Growth and Development Research Center, Research Institute for Primordial Prevention of Non-Communicable Disease, Isfahan University of Medical Sciences, Isfahan, Iran; 3 Faculty of Medical Sciences, Department of Medical Genetics, Tarbiat Modares University, Tehran, Iran; 4 Skull Base Research Center, The Five Senses Health Institute, Rasoul Akram Hospital, Iran University of Medical Sciences, Tehran, Iran; 5 Department of Neurology, Rasoul Akram Hospital, Iran University of Medical Sciences, Tehran, Iran; 6 Department of Pediatrics, School of Medicine, Mashhad University of Medical Sciences, Mashhad, Iran; 7 Neuroscience Research Center, Mashhad University of Medical Sciences, Mashhad, Iran; 8 Dr. Farhud’s Genetic Clinic, Tehran, Iran; 9 School of Advanced Medical Science, Islamic Azad University, Tehran, Iran; 10 National Institute of Genetic Engineering and Biotechnology, Tehran, Iran; 11 Department of Neurology, Johns Hopkins Medicine, Baltimore, MD USA; 12 Jefferson Institute of Molecular Medicine, Thomas Jefferson University, Philadelphia, PA USA; 13 Department of Dermatology and Cutaneous Biology, Sidney Kimmel Medical College, Thomas Jefferson University, Philadelphia, PA USA; 14 Pediatric Neurology Division, Children’s Medical Center, Pediatrics Center of Excellence, Ataxia Clinic, Tehran University of Medical Sciences, Tehran, Iran

**Correction : Orphanet Journal of Rare Diseases (2023) 18:177** 10.1186/s13023-023-02780-9

Following publication of the original article [1], we have been notified that the sentence on equal contribution of the first authors was missed. It should be as follows:

Ali Zare Dehanavi and Maryam Bemanalizadeh have contributed equally to this work.

