# Supplementary material for: Phenotype and genotype heterogeneity of PLA2G6-associated neurodegeneration in a cohort of pediatric and adult patients
Source: Orphanet J Rare Dis. 2023 Jul 5;18:177. doi: 10.1186/s13023-023-02780-9 (PMC10320940; doi:10.1186/s13023-023-02780-9)
Supplement: Supplementary file 1 — Additional file 1. Results of Clustal omega on sequence alignment of the human PLA2G6 protein. [file 13023_2023_2780_MOESM1_ESM.docx]

**A) Results of clustal omega:**

sp|E1BB89 MQEAATRGEVGMGDRARLTRDSVFFQTEGEGGGASPPAPQRMQFFGRLVNTLSSVTNLFV 60

sp|O60733 -----------------------------------------MQFFGRLVNTFSGVTNLFS 19

sp|P97819 -----------------------------------------MQFFGRLVNTLSSVTNLFS 19

**********:*.*****

sp|E1BB89 NPFRVKEVAVEDYHSRRRVREEGQLILFQNSSNRTWDCILVNPRNAQSGFRLFQLETEAD 120

sp|O60733 NPFRVKEVAVADYTSSDRVREEGQLILFQNTPNRTWDCVLVNPRNSQSGFRLFQLELEAD 79

sp|P97819 NPFRVKEVSLTDYVSSERVREEGQLILLQNVSNRTWDCVLVSPRNPQSGFRLFQLESEAD 79

********:: ** * **********:** ******:**.*** ********** ***

sp|E1BB89 ALVNFQQYSSQLPPFYESSTHILQAEVLQQLTDLIRSHPSWSVAHLAVELGIRECFHHSH 180

sp|O60733 ALVNFHQYSSQLLPFYESSPQVLHTEVLQHLTDLIRNHPSWSVAHLAVELGIRECFHHSR 139

sp|P97819 ALVNFQQFSSQLPPFYESSVQVLHVEVLQHLTDLIRNHPSWTVTHLAVELGIRECFHHSR 139

*****:*:**** ****** ::*:.****:******.****:*:***************:

sp|E1BB89 IISCANNKENEEGCTPLHLACRKGDTEVLSELVQHCRANMDVTDNSGETAFHYAVQGDSS 240

sp|O60733 IISCANCAENEEGCTPLHLACRKGDGEILVELVQYCHTQMDVTDYKGETVFHYAVQGDNS 199

sp|P97819 IISCANSTENEEGCTPLHLACRKGDSEILVELVQYCHAQMDVTDNKGETAFHYAVQGDNP 199

****** ***************** *:* ****:*:::***** .***.********.

sp|E1BB89 QVLQLLGKNASGGLNQVNNQGLTPLHLACQLGKQEMVRVLLLCNARCNIMGPGGYPIHTA 300

sp|O60733 QVLQLLGRNAVAGLNQVNNQGLTPLHLACQLGKQEMVRVLLLCNARCNIMGPNGYPIHSA 259

sp|P97819 QVLQLLGKNASAGLNQVNNQGLTPLHLACKMGKQEMVRVLLLCNARCNIMGPGGFPIHTA 259

*******:** .*****************::*********************.*:***:*

sp|E1BB89 MKFSQKGCAEMIVSMDSSQIHSKDPRYGASPLHWAKNAEMARLLLKRGCDVNGTSSAGNT 360

sp|O60733 MKFSQKGCAEMIISMDSSQIHSKDPRYGASPLHWAKNAEMARMLLKRGCNVNSTSSAGNT 319

sp|P97819 MKFSQKGCAEMIISMDSNQIHSKDPRYGASPLHWAKNAEMARMLLKRGCDVDSTSSSGNT 319

************:****.************************:******:*:.***:***

sp|E1BB89 ALHVAVMRNRFDCVMVLLTHGANADARGEHGNTPLHLAMSKDNVEMIKALIVFGAEVDTP 420

sp|O60733 ALHVAVMRNRFDCAIVLLTHGANADARGEHGNTPLHLAMSKDNVEMIKALIVFGAEVDTP 379

sp|P97819 ALHVAVMRNRFDCVMVLLTYGANAGARGEHGNTPLHLAMSKDNMEMVKALIVFGAEVDTP 379

*************.:****:****.******************:**:*************

sp|E1BB89 NDFGETPAFIASRISKLVTRKALLTLLRTVGADYRFPLTQGFSTEQCSAA--APSFSMER 478

sp|O60733 NDFGETPTFLASKIGRLVTRKAILTLLRTVGAEYCFPPIHGVPAEQGSAAPHHP-FSLER 438

sp|P97819 NDFGETPALIASKISKLITRKALLTLLKTVGADHHFPIIQGVSTEQGSAAATHPLFSLDR 439

*******:::**:*.:*:****:****:****:: ** :*. :** *** * **::*

sp|E1BB89 SHPPPISLNNLELQDITQISRARKPAFILSSMRDEKRTHDHLLCLDGGGVKGLVIIQLLI 538

sp|O60733 AQPPPISLNNLELQDLMHISRARKPAFILGSMRDEKRTHDHLLCLDGGGVKGLIIIQLLI 498

sp|P97819 TQPPAISLNNLELQDLMPISRARKPAFILSSMRDEKRSHDHLLCLDGGGVKGLVIIQLLI 499

::** **********: ***********.*******:***************:******

sp|E1BB89 AIEKASGIATKDLFDWVAGTSTGGILALAILHSKSMAYMRGVYFRMKDEVFRGSRPYESG 598

sp|O60733 AIEKASGVATKDLFDWVAGTSTGGILALAILHSKSMAYMRGMYFRMKDEVFRGSRPYESG 558

sp|P97819 AIEKASGVATKDLFDWVAGTSTGGILALAILHSKSMAYMRGVYFRMKDEVFRGSRPYESG 559

*******:*********************************:******************

sp|E1BB89 PLEEFLKREFGEHTKMTDVKKPKVMLTGTLSDRQPAELHLFRNYEAPECVREPRFSQNVN 658

sp|O60733 PLEEFLKREFGEHTKMTDVRKPKVMLTGTLSDRQPAELHLFRNYDAPETVREPRFNQNVN 618

sp|P97819 PLEEFLKREFGEHTKMTDVKKPKVMLTGTLSDRQPAELHLFRNYDAPEAVREPRCNQNIN 619

*******************:************************:*** ***** .**:*

sp|E1BB89 LKPPTHPSEQLVWRAARSSGAAPTYFRPNGRFLDGGLLANNPTLDAMTEIHEYNQDLIRK 718

sp|O60733 LRPPAQPSDQLVWRAARSSGAAPTYFRPNGRFLDGGLLANNPTLDAMTEIHEYNQDLIRK 678

sp|P97819 LKPPTQPADQLVWRAARSSGAAPTYFRPNGRFLDGGLLANNPTLDAMTEIHEYNQDMIRK 679

*:**::*::***********************************************:***

sp|E1BB89 GQDSKVKKLSVVVSLGTGRSPQVPVTCVDVFRPSNPWELAKTVFGAKELGRMVVDCCTDP 778

sp|O60733 GQANKVKKLSIVVSLGTGRSPQVPVTCVDVFRPSNPWELAKTVFGAKELGKMVVDCCTDP 738

sp|P97819 GQGNKVKKLSIVVSLGTGKSPQVPVTCVDVFRPSNPWELAKTVFGAKELGKMVVDCCTDP 739

** .******:*******:*******************************:*********

sp|E1BB89 DGRAVDRARAWCEMVGIQYFRLNPQLGTDIMLDEINDTVLVNALWETEVYIYEHREQFQK 838

sp|O60733 DGRAVDRARAWCEMVGIQYFRLNPQLGTDIMLDEVSDTVLVNALWETEVYIYEHREEFQK 798

sp|P97819|PLPL9_MOUSE DGRAVDRARAWCEMVGIQYFRLNPQLGSDIMLDEVSDAVLVNALWETEVYIYEHREEFQK 799

***************************:******:.*:******************:***

sp|E1BB89 LVQLLLSP 846

sp|O60733 LIQLLLSP 806

sp|P97819|PLPL9_MOUSE LVQLLLSP 807

*:******
